# Reducing Health Inequalities in Aging Through Policy Frameworks and Interventions

**DOI:** 10.3389/fpubh.2020.00315

**Published:** 2020-07-31

**Authors:** Frances A. S. MacGuire

**Affiliations:** Department of Epidemiology and Public Health, University College London, London, United Kingdom

**Keywords:** inequalities, cohort, healthy aging, policy, interventions, proportional universalism

## Abstract

*Lifepath*, a European Commission Horizon 2020 programme of research adopted a life course approach to understanding the impacts of socioeconomic differences on healthy aging and considered the relative importance of lifetime effects by comparing studies on childhood and adult risks. A key component of the programme was the identification of policy relevant results and messages. Longitudinal European cohorts of over 1.7 million individuals from 48 independent cohort studies were harmonized and followed for the key outcomes of mortality and functional decline. Biological markers, allostatic load, and DNA methylation were also examined to help unravel the impact of socioeconomic factors including education, occupation, or income on aging. It is well-recognized that socioeconomic position affects behaviors such as smoking, high alcohol consumption, low physical activity, and a diet low in fruit and vegetables. *Lifepath* indicated that socioeconomic status is an independent risk factor for death and disease but that it also helps drive the uptake of these well-recognized risk behaviors. The evidence from *Lifepath* points to a suite of possible policies, some universal, some targeted but it was not possible to assess specific interventions, other than conditional cash transfers, or to explore how interventions might be effective in reducing health inequalities in aging. Nevertheless, it was clear that the timing of interventions is important as the consequences of early interventions may span the whole life course. These influences have important implications for policy making, since appropriate policies can reverse the embodiment of socioeconomic disadvantage, thus reducing health inequalities and resulting in healthier aging. Applying principles of proportional universalism as one approach to reducing inequalities should be considered.

## Introduction

Healthy aging is an important public health issue, both nationally and internationally. The World Health Organisation (WHO) recognizes healthy aging as a process whereby all people of all ages are able to live a healthy, safe and socially inclusive lifestyle (1). However, it is widely recognized that inequalities experienced from the earliest years of life, and throughout the life course, undermine healthy aging.

Looking beyond the provision of health and social care, the social determinants of health have a major effect on health and well-being. These factors include housing quality, education, social connectivity, climate change, and local environmental damage. Action on these social determinants of health is needed across the life course to reduce inequalities. WHO also promotes a “health in all polices” approach which recognizes that all arms of government can influence the determinants of health and should develop policies which support good health.

Aging can be considered in three component parts: physical (measured as activities of daily living or ADL), mental (measured as cognitive decline), and social (participation in community activities) (2–4).

The number of people aged 60 and over is expected to increase from 901 million to 1.4 billion between 2015 and 2030 (5). Life expectancy increased throughout the second half of the twentieth century but in recent decades this increase has come with more years spent in poor health. It has been estimated, for example, that an English male with a life expectancy of 79.5 years in 2014–16 would have an average healthy life expectancy of 63.3 years, spending around 20 per cent of his life in poor health (6). An English female, with a life expectancy of 83.1 years, would spend 19.2 years (23 per cent) in poor health.

The rapid aging of populations, and the rising numbers of older people living in suboptimal health, highlights the need to develop policies and practices to support healthy aging through the life course and address health inequalities in old age.

As noted, healthy aging includes the maintenance of physical and cognitive functioning, as well as good mental health (3). With increasing age, most people experience a gradual decline in all of these. Despite this decline in health that naturally accompanies aging, many older people lack access to adequate health care (7).

In countries that lack universal health care, at no or low cost, older people may be forced to choose between paying medical costs and other basic needs such as for food, warmth and accommodation. In addition, health care services are not always age-appropriate, particularly in rural areas of low-income countries (8).

However, there are stark differences in healthy aging outcomes between different social groups. Most aging related health outcomes are strongly associated with socioeconomic characteristics of individuals (9, 10). This means that people who have higher education attainment, better jobs, or higher income tend to have better physical or cognitive function compared to those who experience socioeconomic disadvantage.

The impacts of socioeconomic circumstances on healthy aging are now well-documented. People living with socioeconomic disadvantage are more likely to develop disease or die earlier than those living in more advantageous circumstances. This pattern has been described as the social gradient, where the risk of poor health tends to increase with step declines in socioeconomic position (SEP).

The social gradient demonstrates the need for policies and interventions that “level up” health, i.e., raise the health of the worst off to the highest level achievable within society. One approach to responding to this need is “proportional universalism” and is described as policies that are universal and benefit everyone in society, but that are at a scale and intensity that are proportionate to the level of disadvantage (11).

Evidence from longitudinal studies (cohorts) helps us understand a range of trajectories for aging. This evidence can be used to inform policies and interventions to address health inequalities in aging. However, it is less clear which specific policies national and local governments should introduce to reduce the gradient, and the inequalities that it represents, and whether there is sufficient political will to implement such policies.

## Lifepath Studies—Understanding the Role of Health Inequalities on Aging

The European Commission Horizon programme funded *Lifepath*, a life-course approach to understanding the impacts of socioeconomic differences on healthy aging (12). The studies considered under *Lifepath* examined the relative importance of lifetime effects by comparing studies on childhood and adult risks. A key component of the programme was the identification of policy-relevant results and messages.

The WHO recognizes six clear risk factors for unhealthy aging: tobacco use, alcohol consumption, insufficient physical activity, raised blood pressure, obesity, and diabetes. As part of *Lifepath*, researchers explored SEP as a risk factor for adult non-communicable diseases in a multi-cohort study of over 1.7 million individuals from 48 independent cohort studies from the UK, France, Switzerland, Portugal, Italy, the USA, and Australia (13). This work showed that SEP is an independent risk factor for mortality and functional decline, in addition to the risk factors listed above.

Low SEP was associated with 2.1 years of life lost (YLL) between ages 40 and 85 years and was comparable with YLL from the other six risk factors. This finding emphasized the importance of not only focusing on the six risk factors but also on addressing low socioeconomic position. Studies considered under *Lifepath* indicated that not only is socioeconomic status an independent risk factor for death and disease, it also helps drive the six risk factors.

Poorer health over the life course has been associated with early life factors, particularly adverse childhood experiences, and lack of availability of social support (14). In studies of health inequalities among older people, the strongest relationship was found to be between poverty and poor health (15). Furthermore, health inequalities in old age reflect accumulated disadvantage over the life course as well as inequalities experienced at older ages associated with geographic location of residence, gender, and ageist attitudes and practices (16).

Extensive existing evidence implies that to reduce health inequalities at older ages, policies, and interventions need to address social determinants of health in early life and across the life course. The consequences of early interventions may span the whole life course with important implications for policy-making. However, older people carry the burden of ill-health. Strategies to tackle inequalities in healthy aging must also address social inequalities experienced at older ages.

Older people at the lower end of the social gradient often have more difficulty in accessing health services even though they are already likely to experience poorer health (8). Examples include Nazroo (17), in a study of 12 European countries who observed inequalities, by education level, among people aged 50 and over in visits to medical specialists and dentists. In the UK, older people in lower SEP groups had less access to health services such as mammography screening, vaccinations, eye and dental exams, and heart surgery (18). People living in the most deprived areas of Scotland were diagnosed with more than one condition 10–15 years earlier than those living in the least deprived areas (19).

Policies can be implemented to influence health determinants including access to health and social care, risk behaviors such as smoking and physical inactivity, and health literacy which should support people to enter old age in good health. Such policy responses should be directed at people living in poverty and other disadvantaged groups. Psychosocial aspects such as social engagement have also been identified as supporting healthy aging (20).

## Strategies and Policies to Reduce Inequalities in Aging

While there is strong evidence now that inequalities in health are influenced both by socioeconomic circumstances and risk behaviors, evidence-based policies for reducing these inequalities are, not at first sight, so obvious. A plethora of strategies and objectives exists but only a limited number of specific policies and practices to reduce inequalities have been described in the literature, partly because it is hard to experiment with policy on populations, both practically and ethically.

The WHO Global Strategy and Action Plan on Aging and Health (GSAP) includes five strategic objectives (1):

The GSAP recognizes that healthy aging takes place across the entire life course. Consequently, policies and interventions can be designed and implemented at different life stages to impact the trajectory of healthy aging.

Since low SEP has strong effects on aging, socioeconomic circumstances should be included in local and global health strategies, health risk surveillance, interventions, and policies to reduce health inequalities throughout the life course. Improving socioeconomic circumstances may also reduce the uptake of behavioral risk factors.

A number of systemic policies improve socioeconomic circumstances, for example, free health care at the point of need, compulsory education, income tax credits, and requirements for safe school and work environments. Human rights and anti-discrimination legislation also affect health inequalities, along with employment and housing laws.

Policy and interventions in early childhood should be seen as part of a comprehensive strategy to reduce health inequalities in later life. However, policies and interventions are also needed specifically to support health in later life. In one study, the provision of more generous minimum pensions and higher expenditure on social care for the elderly, resulted in reduced health inequalities in the age group 65–80 years (21). In this way welfare policies can moderate the association between SEP and health. This finding reflects analysis from the European Office of WHO which identified six policies with statistically significant potential to reduce short term health inequalities (22):

- Increasing public expenditure on housing and community amenities.
- Increasing expenditure on labor market policies.
- Reducing income inequality.
- Increasing social protection expenditure; reducing unemployment.
- Reducing out-of-pocket payments for health.

Policies may also be designed to tackle ageism, as highlighted in the GSAP objectives in Box 1 above, which should help to reduce inequalities in employment practices for older people and access to certain healthcare interventions such as screening, surgery, and transplants. In the UK, for example, the Public Sector Equality Act aims to address ageism through a duty for public agencies to consider and apply fairness and equality in making decisions and developing policies or services (23).

Box 1 **The WHO Global Strategy and Action Plan on Aging and Health (GSAP) includes five strategic objectives (1).**

1. **Commitment to action on healthy aging in every country**
  1.1 Establish national frameworks for action on healthy aging
  1.2 Strengthen national capacity to formulate evidence-based policy
  1.3 Combat ageism and transform understanding of aging and health
2. **Developing age-friendly environments (environments in the broadest sense, including physical, social, and policy environments)**
  2.1 Foster older people's autonomy
  2.2 Enable older people's engagement
  2.3 Promote multisectoral action
3. **Aligning health systems to the needs of older populations**
  3.1 Orient health systems around intrinsic capacity and functional ability
  3.2 Develop and ensure affordable access to quality older person-centered and integrated clinical care
  3.3 Ensure a sustainable, appropriately trained, and managed workforce
4. **Developing sustainable and equitable systems for long-term care**
  4.1 Establish and continually improve a sustainable and equitable long-term care system
  4.2 Build workforce capacity and support caregivers
  4.3 Ensure the quality of person-centered and integrated long-term care
5. **Improving measurement, monitoring, and research for healthy aging**
  5.1 Agree on ways to measure, analyse, and monitor healthy aging
  5.2 Strengthen research capacities and incentives for innovation
  5.3 Research and synthesize evidence on healthy aging

While broad, systemic policies such as good pension provision and access to health care should be effective in all countries, some specific policies that are aimed at certain populations may work better in some countries than others. As noted, relatively few polices aimed at addressing health inequalities in aging have been tested experimentally.

One approach to increasing SEP to improve health early in the life course is the policy intervention of “conditional cash transfers (CCT).” Popular in low- and middle-income countries, they have been used infrequently in Europe and the U.S. CCT programmes aim to reduce short-term poverty and to break intergenerational poverty by providing a cash sum to people on low income in exchange for the pursuit of positive health behaviors. They are often designed around child health and might include vouchers for breastfeeding or support for attendance at vaccination clinics (24).

The results from CCT programmes are mixed. While such programmes affect specific behaviors being promoted, it is not clear whether that they result in more fundamental changes which could deliver better child health (25).

Social prescribing—where GPs and other health professionals “prescribe” sessions at the gym or other activity based groups (dance, yoga, time in high quality natural environments)—is more applicable to adults as this is an approach that promotes better self-care rather than supporting care-givers, such as parents, which CCT aims to support. Social prescribing is used as a complementary activity to medication, often for vulnerable people with multiple health and social needs and aims to alleviate social isolation and increase physical activity in older age (26).

While interventions are needed throughout the life course, current older generations need specific support to reduce health inequalities. Resources are needed to reduce poverty in old age, provide disability support and care at home, and high-quality residential care. A growing elderly population may result in an increased health burden of dementia and more people requiring residential care. Such care may become a necessary option for more families with older relatives with advanced dementia, even if it is not a route that family members really want to take. Family members must be confident that their elderly relatives will be cared for safely and with dignity in their final years. Examples of woefully inadequate care which have been reported by the media highlight this is not always the case. Health and social care for older people needs sufficient investment, skilled staff and integration between services.

Many existing policies and interventions will help reduce inequalities in aging although they may not have been designed initially to do this. As an example, Table 1 shows some of the existing relevant policies and interventions in the UK (27). Other countries across Europe will have similar measures. The EU and UN Economic Commission for Europe (UNECE) created an online Active Aging Index to help inform policy making, for example European Commission (28). The AAI is a composite measure which aggregates scores from four domains: (a) employment; (b) participation in society; (c) independent, healthy, and secure living; and (d) enabling environment.

**Table 1:** Existing and proposed universal and targeted health and social care policies or interventions for four age groups which reduce health inequalities (Early Years 0–4, Childhood 5–18, working age 19–66, and 67 years+) in the UK (27).

	**Early years 0–4**	**Childhood 5–18**	**Working age 19–66**	**67 years +**
**Universal**	Universal health care	Universal health care	Universal health care	Universal health care
	Child benefit (UK)	Child benefit (UK)		State pension
	Immunization	Immunization		Immunization e.g., fluWinter FuelPayments
		Universal education		
			Health and safety/Occupational health	
	Smoke free public places	Smoke free public places	Smoke free public places	Smoke free public places
	Sugar tax	Sugar tax	Taxes on alcohol, tobacco, and sugar	Taxes on tobacco, alcohol, and sugar
	Ban on hydrogenated trans fats	Ban on hydrogenated trans fats	Ban on hydrogenated trans fats	Ban on hydrogenated trans fats
	Food labeling – calories, traffic lights	Food labeling – calories, traffic lights	Food labeling – calories, traffic lights	Food labeling – calories, traffic lights
	Promote active transport (walking, cycling)	Promote active transport (walking, cycling)	Promote active transport (walking, cycling)	Promote active transport (walking, cycling)
**Targeted at low SEP**			Conditional cash transfers	Flexible working practices which make work more attractive than retirement
	Provision of suitable housing (space and free of damp and pollution)	Provision of suitable housing (space and free of damp and pollution)	Provision of suitable housing (space and free of damp and pollution)	Provision of suitable housing (space and free of damp and pollution)
	Residential care (children/young people)	Residential care (children/young people)	Residential care (adult)	Residential/nursing care (elderly)
			Universal credit (UK)	
	Emergency support during recessions	Emergency support during recessions	Emergency support during recessions	Emergency support during recessions

The four domains were considered in more detail as:

- Encouraging working lives and maintaining work ability.
- Promoting participation, non-discrimination, and the social inclusion of older persons.
- Promoting and safeguarding dignity, health, and independence in older age.
- Maintaining and enhancing intergenerational solidarity.

**Figure 1:**
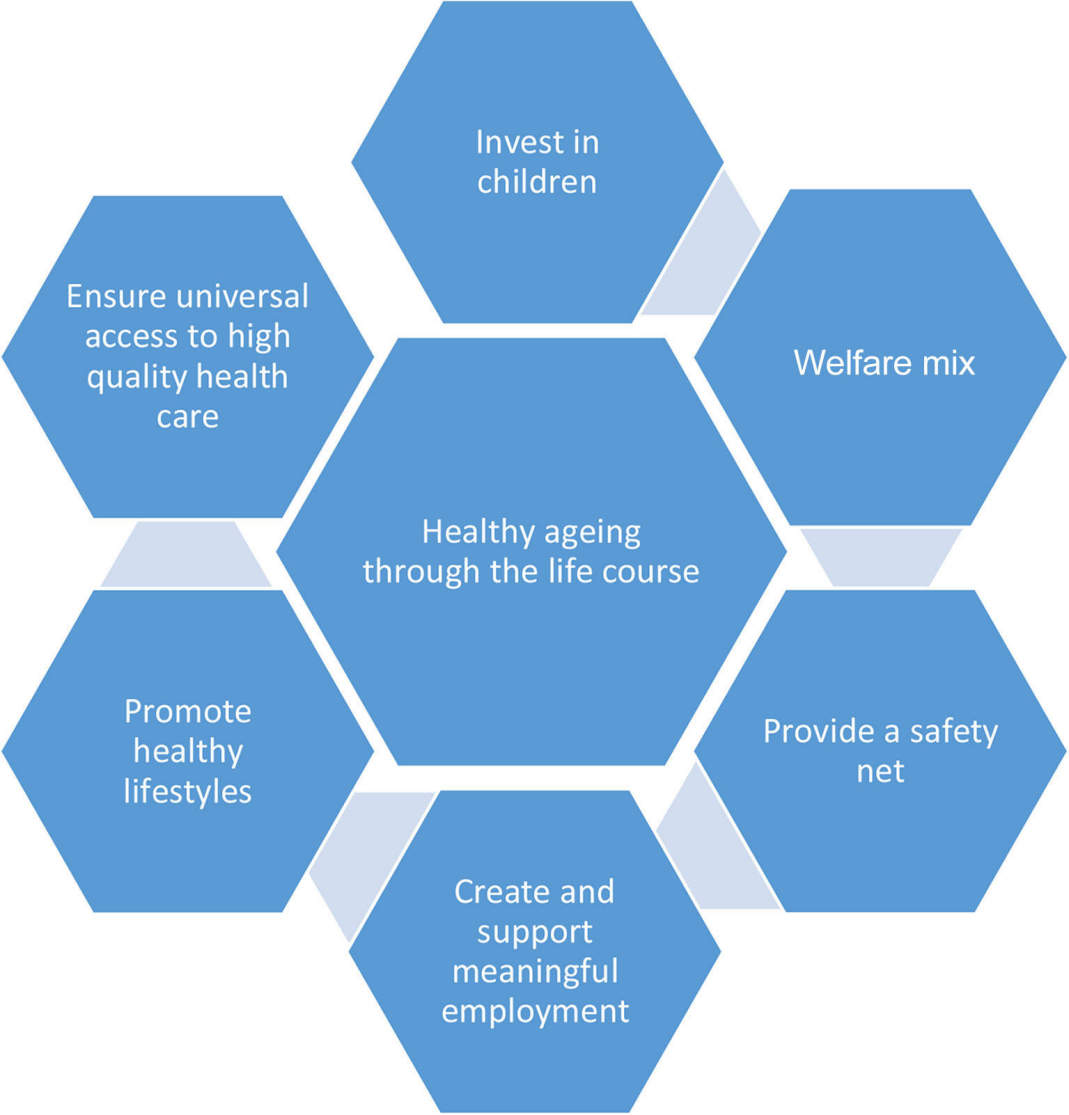
Strategies and policy areas that reduce health inequalities throughout the life course [adapted from (29)].

Policies and interventions which support healthy aging through the life course can be grouped into six areas—investing in children, welfare support, provision of a safety net, creating meaningful employment, healthy lifestyles, and universal health care (see Figure 1). Importantly, promoting healthy lifestyles is only one of these strands and yet so much policy effort focuses on these behaviors. While reducing individual behavioral risks is important, systemic change is also needed to reduce health inequalities, particularly in access to healthcare, provision of childcare for pre-school children and social care (help with feeding, dressing, housework and maintenance, reducing isolation). Broader policies that protect and enhance local communities and environments which underpin sustainable planning will also help reduce health inequalities. Such an approach was recommended by the Marmot Review in the UK which identified six policy objectives in addressing health inequalities (11):

- Give every child the best start in life.
- Enable all children, young people, and adults to maximize their capabilities and have control over their lives.
- Create fair employment and good work for all.
- Ensure a healthy standard of living for all.
- Create and developing sustainable places and communities.
- Strengthen the role and impact of ill-health prevention.

## Commissioning Services for Older People

In order to explore possible polices and interventions to support healthy aging, another EU Horizon research project—ATHLOS^1^—has undertaken consultation with stakeholders as part of its research programme.

In the first wave of consultation, participants completed an online survey on factors identified as important for healthy aging among people aged 50+. This survey was part of a systematic review conducted by the ATHLOS consortium and from the wider literature (30). The online survey compiled 310 replies across Europe, of which 145 people were aged 50+. The top-five factors identified by the stakeholders as the most important influences on healthy aging for people aged 50+ were as follows:

- Physical activity (58.6% of the total sample).
- Access to preventive, diagnostic, and health care services (53.8%).
- Adequate income (51.0%).
- Participation in community and/or social activities (51.0%).
- Adequate and affordable housing (45.5%).

When asked about the top-five factors that should be prioritized by policy-makers to enable older people (50+) to live a healthy life and keep on doing what they want to do, the majority of respondents replied with the following:

- Access to preventive, diagnostic, and health care services (64% of those 145 respondents aged 50+).
- Adequate income (53%).
- Providing opportunities to participation in community and/or social activities (48%).
- Access to adequate social care services (46%).
- Access to safe and suitable transport and mobility options (41%).

As a comparison, in the UK, the social care Green Paper lists seven key outcomes (23):

- Improved health and emotional well-being.
- Improved quality of life.
- Making a positive contribution.
- Increased choice and control.
- Freedom from discrimination or harassment.
- Economic well-being.
- Maintaining personal dignity and respect.

In the UK the National Service Framework (NSF) outlines the evidence base for a range of health promotion activities for older people. The strongest evidence found was for increased physical activity, improved diet and nutrition, and immunization programmes for influenza (31). The importance of older people being able to access population-wide health promotion initiatives (such as smoking cessation) and initiatives to reduce poverty through benefits advice and support were also emphasized.

In the UK, specific care needs at the individual level are detailed in the Prevention Package for Older People (32). This was published as a series of resources to support commissioning of services for older people such as addressing falls, foot care, hearing services, intermediate care, and discharge from hospital. Forthcoming resources are planned on depression, continence, and arthritis.

## Conclusion

The impact of socioeconomic circumstances on health inequalities in aging is clear. There are several well-evidenced strategies for reducing inequalities in aging, including increased access to health services and adequately-funded pension schemes. While few policies have been tested experimentally, we know that key systemic strategies such as universal health care and education, as well as welfare and employment support, are effective in reducing health inequalities. We also know that reducing individual and population behavioral risks supports healthy aging but we are less clear about the most effective policies and programmes to reduce those risks. Taking physical activity, as one example: how should government invest public money to promote, encourage, and support individuals and populations to be more active? Options include supporting active travel (walking, cycling) and social prescribing but barriers exist to uptake and more evidence of effective policy is needed.

A key area of concern is that support and funding for systemic policies and initiatives that are known to reduce inequalities are waning in some countries in Europe. This is happening despite evidence that fairer societies do better on a range of indicators and that some population-based, systemic polices are more effective than programmes targeting individuals. Societal investment is needed in early and middle years, as well as in older age, to support healthy aging and to reduce inequalities. Ultimately this is beneficial to individuals and society.

## Data Availability Statement

Data discussed in the paper is available through the EU Lifepath and ATHLOS programmes, contact p.vineis@imperial.ac.uk and m.bobak@ucl.ac.uk.

## Ethics Statement

Ethical review and approval was not required for the study on human participants in accordance with the local legislation and institutional requirements. The patients/participants provided their written informed consent to participate in this study.

## Author Contributions

The author confirms being the sole contributor of this work and has approved it for publication.

## Conflict of Interest

The author declares that the research was conducted in the absence of any commercial or financial relationships that could be construed as a potential conflict of interest.

## Footnotes

^1^ATHLOS – Aging Trajectories of Health – Longitudinal Opportunities and Synergies.
